# Quantifying the Spatial Footprint of Agriculture‐Driven Edge Effects in a Global Deforestation Hotspot

**DOI:** 10.1111/gcb.70737

**Published:** 2026-02-10

**Authors:** Sebastián Torrella, Matthias Baumann, Marie Pratzer, Sebastián Aguiar, María Piquer‐Rodríguez, Rubén Ginzburg, Gregorio Gavier Pizarro, Tobias Kuemmerle

**Affiliations:** ^1^ Geography Department Humboldt‐Universität Zu Berlin Berlin Germany; ^2^ Departamento de Ecología, Genética y Evolución, Facultad de Ciencias Exactas y Naturales Universidad de Buenos Aires and Instituto de Ecología, Genética y Evolución de Buenos Aires (IEGEBA) Buenos Aires Argentina; ^3^ Integrative Research Institute for Transformations of Human‐Environment Systems (IRI THESys) Humboldt‐Universität Zu Berlin Berlin Germany; ^4^ Cátedra de Dasonomía, Departamento de Producción Vegetal, Facultad de Agronomía Universidad de Buenos Aires Buenos Aires Argentina; ^5^ Laboratorio de Análisis Regional y Teledetección, IFEVA, Facultad de Agronomía CONICET Buenos Aires Argentina; ^6^ Institute of Geographical Sciences Freie Universität Berlin Berlin Germany; ^7^ Instituto de Fisiología y Recursos Genéticos Vegetales (IFRGV)—CIAP—INTA Córdoba Argentina

**Keywords:** aboveground biomass, agricultural expansion, Bayesian multilevel models, dry Chaco, shrub cover, tree cover, tropical dry forests

## Abstract

Tropical dry forests are under high and rising pressure from agricultural expansion, resulting in widespread forest conversion and fragmentation. Additionally, remaining forests experience a range of edge effects through agriculture once it has been established, yet such agriculture‐driven edge effects remain weakly understood. Focusing on the Argentine Dry Chaco, a global hotspot for deforestation, we utilized satellite‐based forest structure indicators within a Bayesian Hierarchical Modelling framework to quantify and map agricultural edge effects on fractional tree and shrub cover, and aboveground biomass. Specifically, we assessed how far edges reach into forests away from the forest‐agriculture interface, whether edge effects differ among post‐deforestation land uses (i.e., cropping vs. ranching), and how edge effects evolve over time. We reveal large agriculture‐driven edge effects in the Chaco, penetrating > 700 m into adjacent forests, with reductions of up to 41% in our structural parameters. Cropping was associated with much stronger edge effects than pastures, likely due to the combined effect of environmental (e.g., wind) and management (e.g., pesticide drift) factors, while silvopastures had much lower edge effects. Projecting our models across the region showed that 18% of remaining forests are degraded, with an estimated total loss of 92.3 million tons of aboveground biomass. Lastly, agriculture‐driven edge effects intensified for long periods after initial deforestation (e.g., > 30 years for tree cover), suggesting agricultural expansion creates a degradation debt that unfolds over decades. We conclude that agriculture‐driven edge effects are a major, yet often overlooked, consequence of agricultural expansion, leading to profound degradation far beyond the deforestation footprint. Despite their importance, these effects remain systematically underestimated in sustainability analyses, such as carbon accounting or biodiversity impact assessments. Our work supports views that conservation planning should prioritize large and contiguous forest patches to help maintain ecologically functional forests.

## Graphical abstract

Tropical dry forests face increasing pressure from agricultural expansion, but how this expansion impacts on remaining forests is unclear. We quantified and mapped agricultural edge effects on tree cover, shrub cover, and above‐ground biomass in the Argentine Chaco, a global deforestation hotspot. Agriculture caused strong edge effects, especially for cropping. Edge effects intensified for decades after deforestation, creating a long‐lasting degradation debt. Overall, we estimate that edge effects have degraded 18% of remaining forests. Our results show that agricultural expansion drives substantial, often overlooked degradation far beyond the deforestation footprint.

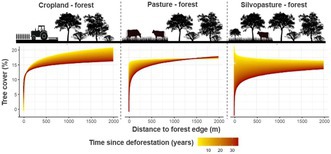

## 1 Introduction

Tropical dry forests and woodlands account for nearly 40% of all tropical forests, harbor high biodiversity, and contribute 30% of terrestrial primary productivity (Miles et al. 2006). These forests also underpin the livelihoods of millions of people and provide globally relevant ecosystem services, including moisture recycling and carbon storage (Powers et al. 2018). Despite their relevance and conservation value, tropical dry forests remain neglected by science, policymaking, and conservation planning compared to rainforests (Schröder et al. 2021; Rivas and Navarro‐Cerrillo 2024). At the same time, dry forests are under high pressure, particularly from agricultural expansion, leading to a staggering loss of over 71 Mha globally since 2000 (Buchadas et al. 2022). Additionally, agricultural expansion impacts remaining forests in major ways by altering their spatial configuration (Rivas and Navarro‐Cerrillo 2024). Formerly large and contiguous forest tracts become fragmented, leading to the loss of core forest and the creation of many small and often isolated forest patches (Taubert et al. 2018; Ma et al. 2023). Forest fragmentation due to agricultural expansion has been increasing particularly in the tropics, and today 80% of all tropical dry forests are within 500 m of a forest edge (Stan et al. 2024). Understanding how the agriculture/forest interface influences remaining forests is therefore important.

Edge effects, which refer to changes in forest structure and functioning that occur at forest edges (Bourgoin et al. 2024), are a potentially key, but often overlooked, aspect through which agricultural expansion impacts forests. Such edge effects typically are strongest at the immediate forest edge but can extend far from this edge into interior forest patches, with penetration depth referring to the maximum distance at which edge effects are noticeable (Harper et al. 2005). Noticeable edge effects in the tropics have been quantified recently (Bourgoin et al. 2024), though rarely for dry forests. For example, carbon stocks and aboveground biomass in tropical rainforests are about 25% lower at the forest edge (Ordway and Asner 2020; Bauer et al. 2024) but penetration depths reaching up to 2 km (Chaplin‐Kramer et al. 2015). In dry forests, a decrease in stem densities near forest edges has been observed in Madagascar (Malcolm et al. 2017), whereas shifts in species composition—but not in forest structure—have been documented away from edges in Mexico (Benítez‐Malvido et al. 2014). Globally, rainforest canopy height is 25%–40% lower (Nguyen et al. 2023) and 20% lower (Bourgoin et al. 2024), with edge effects penetrating up to 1.5 km into the interior forest (Bourgoin et al. 2024). While this research shows that edge effects are important drivers of tropical forest degradation, an assessment of how agriculture causes edge effects is missing.

Quantifying agriculture‐driven edge effects is challenging for at least three main reasons. First, the type of land use replacing forests likely impacts the magnitude and penetration distance of edge effects (Ribeiro et al. 2019; Willmer et al. 2022; Fletcher Jr et al. 2024). For instance, edge effects due to wind are likely stronger where tree‐less agriculture replaces forests, as opposed to agroforestry systems. In addition, herbicide drift from croplands may affect adjacent forests substantially (Cederlund 2017), or management fires can escape from pastures and impact nearby forests (Cano‐Crespo et al. 2015). However, despite the diverse ways in which post‐deforestation land use could affect edges, no study has systematically examined this relationship in any tropical forest region (Tavares et al. 2019; Franklin et al. 2021).

Second, edge effects due to agricultural expansion likely vary over time (Haddad et al. 2015). Where agriculture expands into natural tropical forests, edge effects should be small right after the expansion yet can be expected to increase over time (Gascon et al. 2000; Laurance et al. 2018). Prior work corroborates this. For instance, in Amazonian forests, carbon stocks at the forest edge decreased during the first 6–8 years after edge creation (Silva Junior et al. 2020). Similarly, edge effects were found to intensify over a 30‐year time period for global tropical rainforests, with no sign of recovery (Bourgoin et al. 2024). Overall, however, changes in the magnitude and penetration depth of edge effects remain weakly understood (Franklin et al. 2021), and we are not aware of a study that has assessed this for tropical dry forests.

Third, edge effects vary depending on the functional or structural parameter used to quantify the edge effect. For instance, agricultural expansion might affect nearby forests more in terms of overall biomass or canopy complexity and height than just tree cover (Laurance et al. 2002). As a result, analyzing only tree cover might underestimate edge effects. Additionally, different post‐deforestation land uses, as well as time since edge creation, likely impact structural parameters differently. For example, herbicide drift from croplands affects life forms and life stages differently, with the strongest effects on herbaceous species and early development stages (Ferreira et al. 2023). Similarly, management fires on pastures can affect both herbaceous and woody vegetation if they escape into nearby forests, but large trees can survive fires better than other plants (Vidal‐Riveros et al. 2023). This leads to substantial complexity when analyzing agriculture‐driven edge effects in tropical forests.

While spatially and temporally detailed data on forest and tree‐cover loss are now available (Hansen et al. 2013; Vancutsem et al. 2021), analyzing agriculture‐driven edge effects in the tropics has been hampered by missing time series on post‐deforestation land use and structural parameters beyond tree cover. Recent advances in remote sensing now allow to reconstruct post‐deforestation land uses in detailed ways (Zalles et al. 2021; Baumann et al. 2022), as well as to map key structural forest parameters such as tree height (Potapov et al. 2021; Qi et al. 2025) or aboveground biomass (Wang et al. 2024) at unprecedented spatial resolution. Yet as the latter often relies on radar sensors that only recently have become operational (e.g., Sentinel‐1) or lidar data that has only been collected for single points in time, these structural parameters cannot be reconstructed back in time. Space‐for‐time substitutions (Blois et al. 2013; Lovell et al. 2023) can be a promising way forward in such situations, as they can approximate the impact of an intervention (i.e., edge creation) by comparing the outcome (i.e., contemporary forest structure) across locations with different timing since this intervention occurred. Integrating space‐for‐time setups in a multilevel modeling framework can furthermore disentangle varying magnitudes and trajectories of edge effects across fundamentally different situations, such as different post‐deforestation land uses (de Marzo et al. 2023).

Here, we adopt such an approach to carry out the first comprehensive, regional‐scale assessment of agriculture‐driven edge effects in tropical dry forest structure. We focused on the Argentinean Dry Chaco, a global deforestation hotspot (Hansen et al. 2013; Buchadas et al. 2022). Agriculture expansion in the region has led to the loss of 19.3 Mha since 1985, equivalent to 28% of the forest cover at that time (Baumann et al. 2022), as well as the widespread fragmentation of the remaining forest (Piquer‐Rodríguez et al. 2015; de la Sancha et al. 2021). Specifically, we asked three research questions:

1. What is the magnitude and penetration depth of edge effects on forest structure (i.e., tree cover, shrub cover, aboveground biomass) away from agricultural areas?
2. How does edge‐effect magnitude vary among different post‐deforestation land uses?
3. How do edge effects vary in relation to the time since agriculture was established?

## 2 Methods

### 2.1 Study Area

**FIGURE 1:**
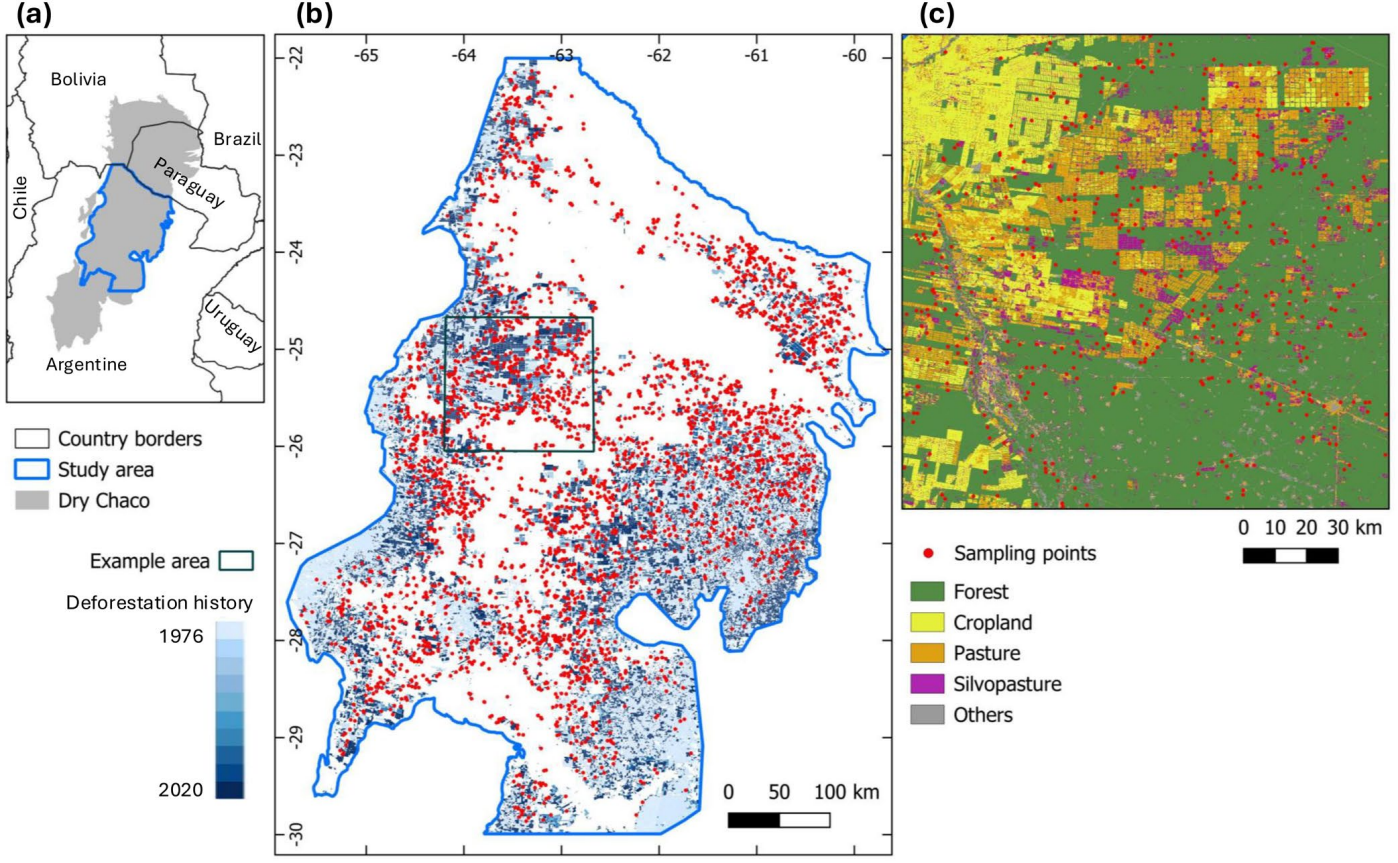
Dry Chaco ecoregion in South America and our study area in Argentina (a); the time when deforestation happened and sampling points allocation (b); and a zoom‐in on an example area in the western Argentine Chaco (c). Map lines delineate study areas and do not necessarily depict accepted national boundaries.

We examined edge effects associated with agricultural expansion in the Argentine Dry Chaco (Morello 2012). Our study area comprises 31.2 million hectares, of which 9.5 million have been deforested since 1975 (Figure 1). Mean annual temperature in the study area ranges from 18°C to 24°C, with a distinct dry and wet season and a strong rainfall gradient from the east (1000 mm) to the west (600 mm); the terrain is predominantly flat, and the dominating soils are *mollisols* and *alfisols* (Morello 2012). The prevailing vegetation community is *xerophilus* forest, mostly dominated by *Schinopsis lorentzii* and *Aspidosperma quebracho‐blanco* (Prado 1993). The landscape also contains other forest types (e.g., riparian forest) associated with active or abandoned rivers, and natural grasslands, mainly in historical riverbeds (Adamoli et al. 1990; Andrade‐Díaz et al. 2023).

Although agriculture has a long history in the Chaco, with waves of settlements of actors practicing various forms of small‐scale cropping and ranching, the large‐scale expansion of industrialized agriculture has mostly happened since the late 1990s. This expansion has been driven primarily by the production of soybean, maize, and wheat, as well as cattle production in cow‐calf systems based on sown pastures (mainly *Panicum maximum* ) and silvopastoral systems (Fernández et al. 2020; Baumann et al. 2022).

### 2.2 Conceptual and Analytical Framework

**FIGURE 2:**
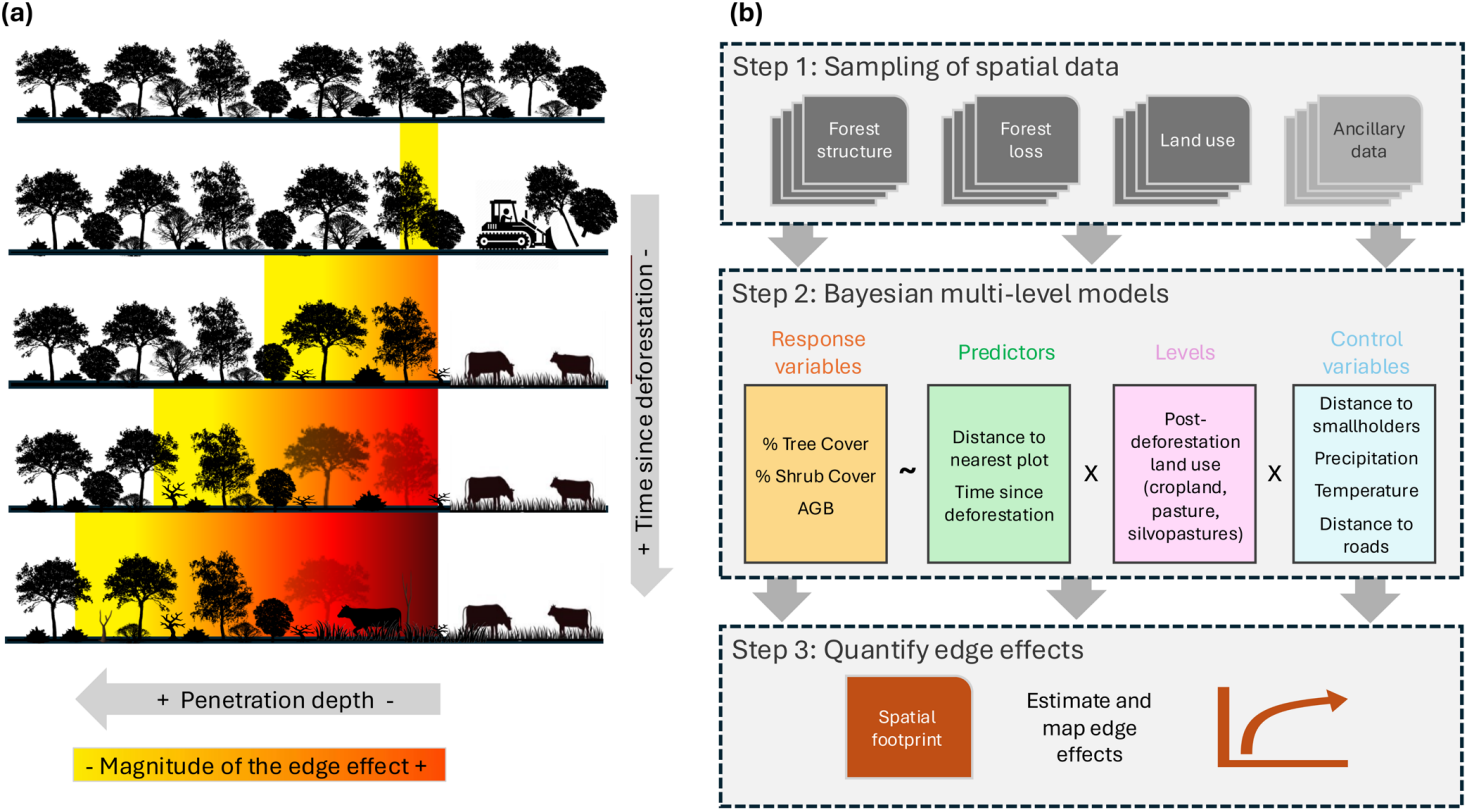
Conceptualization of agriculture‐driven edge effects (a) and analytical framework (b) to analyze their impacts on tropical dry forest structure in the Chaco.

To analyze agriculture‐driven edge effects, we established a conceptual framework assuming that agricultural expansion into forests impacts the structure and composition of the remaining, adjacent forest until a maximum distance of 2000 m (Chaplin‐Kramer et al. 2015). Over time, edge effects can become stronger, both in terms of magnitude and penetration depth (Figure 2a). We translated this conceptual framework into an analytical framework (Figure 2b), consisting of three main steps: (1) sampling random locations inside forest areas and summarizing variables describing forest structure (fractional of tree and shrub cover, aboveground biomass), the deforestation context (distance to deforestation plots, post‐deforestation land use in the nearest plot), as well as a set of control variables that might affect the forest structure (e.g., rainfall, temperature, distance to roads) for these locations; (2) analyzing these data in a multilevel Bayesian framework to quantify the relationship between forest structure and distance to edge, while accounting for possible variation in this relationship due to time since deforestation and type of post‐deforestation land use; and (3) analyzing and mapping the area affected by edge effects.

### 2.3 Sampling Design

To map and characterize deforested areas in the Chaco, we used two Landsat‐based datasets: (1) a time series of manually digitized deforestation plots for the period 1976–2020 (Vallejos et al. 2015, updated annually on http://monitoreodesmonte.com.ar), and (2) a time series of land‐cover maps, including forest, cropland, grassland, and mixed/disturbance areas (Baumann et al. 2022). We combined these time series by assigning to each plot that was deforested based on (1) the dominant land‐cover type as mapped by (2). We labeled plots dominated by cropland or grassland pixels as cropland and pasture, respectively. We labeled as silvopastoral areas all plots identified as deforested but still containing some level of tree cover afterwards. We visually inspected a large set of such plots in very high‐resolution satellite imagery available in Google Earth. This confirmed that these plots contained remnant, scattered large trees, and many showed other signs of ranching use (e.g., fencing, watering points for cattle). This supported the interpretation of Baumann et al. (2022), to label such plots as silvopasture. We also note that this aligns with considerable field knowledge that the author team has from 10+ years of working in the region.

Next, we randomly sampled 3000 points in remaining forest areas with a maximum distance of 2000 m to any deforested plot, assuming this to be the maximum edge effect distance (Chaplin‐Kramer et al. 2015). We excluded areas within 10 km of the study region boundary to ensure that all observations were inside the Dry Chaco (i.e., excluding transitions to other ecoregions). We then recorded for each sampling location (1) the distance to the nearest deforestation plot, (2) the year of deforestation of that plot, and (3) the contemporary land use of that plot (i.e., cropland, pasture, or silvopasture).

### 2.4 Modeling Approach

To assess the impact of agriculture on remaining forests away from deforested plots, we analyzed three response variables describing forest structure: (a) fractional tree cover (i.e., cover of trees > 10 m height), (b) fractional shrub cover (i.e., short trees and large shrubs < 10 m), and (c) aboveground biomass. Tree and shrub cover estimates were derived from Baumann et al. (2018), who implemented a gradient‐boosted regression framework to model these forest structural attributes at a 30‐m spatial resolution for the year 2020, using Landsat‐8 optical and Sentinel‐1 SAR data. The models showed high to moderate predictive power (85.5% for tree cover and 68.5% for shrub cover). Aboveground biomass estimates were obtained from Pötzschner et al. (2022), who also applied a gradient‐boosting approach, combining MODIS and Sentinel‐1 SAR inputs to generate multi‐model predictions at a ~250 m resolution for the year 2019. These models also performed well (*R* ^2^ of 0.89; RMSE = 15.1 t/ha).

We used a Bayesian multilevel model to investigate the relationship of our forest structure variables with the distance to the nearest agriculture/forest edge, as well as how this relationship varied over time and for different post‐deforestation agricultural land uses. Multilevel models (also referred to as hierarchical or varying‐effects models) partition the total variance occurring in a statistical phenomenon into variation at the point vs. variation among broader categories (i.e., levels) (McElreath 2020). In our case, we used the three post‐deforestation land uses (cropland, pasture, and silvopasture) as levels. We chose a multi‐level setup over including them as a categorical predictor for three main reasons. First, our data can be considered nested within these land‐use categories, and a multi‐level model explicitly acknowledges this nested structure, leading to more accurate inference (McElreath 2020). Second, we expect post‐deforestation land uses to have significant variability in their effects on forest structure. By estimating a distribution of effects rather than a single fixed effect for each category, a multi‐level model can account for these variations more effectively (McElreath 2020). Third, a multi‐level model performs partial pooling, resulting in shrinkage of the estimates towards the overall mean, which is beneficial to avoid overfitting (Pratzer et al. 2023).

In addition to the distance to the nearest deforestation plot, we used a range of control factors possibly influencing forest structure. First, we accounted for environmental variation by including mean annual temperature and rainfall from the CHELSA dataset (Karger et al. 2023). Second, we derived the distance to roads and smallholder homesteads inside the forest matrix (Levers et al. 2021), as more accessible forests are likely more used, for instance, to extract firewood or valuable trees (Baumann et al. 2018). Third, we accounted for the possibility that a location might not only be impacted by the closest deforestation plot, but also by other nearby plots that had been deforested before (but which are located farther away). Such a situation could lead to a legacy effect of previous deforestation. We thus calculated, for each point, the distance to the closest deforestation plot for each year 1976–2020 (i.e., for which we had deforestation data available), resulting in a per‐point time series of distances to the nearest deforested plot. We then calculated, for each point, the area under this distance‐time curve. Points that have been close to other deforested areas for a long period show a small area under this distance‐time curve, whereas points that were far away from deforested plots over longer periods and only became close to deforestation recently, as frontiers advanced, have larger areas under this curve. All variables were standardized to zero mean and unit standard deviation before entering the model.

We modeled the three forest structure variables (tree cover, shrub cover, aboveground biomass) separately. To capture non‐linear edge effects, we included logarithmic terms, thus enabling the model to fit non‐linear relationships to distance to forest edge and time. We specified the models as follows:

$${\mathrm{FS}}_{i}\sim N\left(\mu_{i}+\sigma_{i}\right)$$

$$\begin{array}{c} \mu =\alpha_{\mathrm{LC}\left[i\right]}+\beta_{\text{Distance},\mathrm{LC}\left[i\right]}{\text{Distance}}_{i}\times \beta_{\mathrm{Age},\mathrm{LC}\left[i\right]}{\mathrm{Age}}_{i}\\ \;+\beta_{\text{Temp}}{\text{Temp}}_{i}+\beta_{\text{Precip}}{\text{Precip}}_{i}+\beta_{\text{Dist}\_\text{Time}}\text{Dist}\_{\text{Time}}_{i}\\ \;+\beta_{\text{Dist}\_\text{Home}}\text{Dist}\_{\text{Home}}_{i}+\beta_{\text{Dist}\_\text{Roads}}\text{Dist}\_{\text{Roads}}_{i} \end{array}$$

$$\sigma_{i}=T\;\left(3,0,1\right)$$

where forest structure (FS), as the response variable for every point *i*, is predicted as a function of distance to the nearest edge (*Distance*) and edge age (*Age*) with land‐cover (*LC*) level varying effects. Annual mean temperature (*Temp*), annual mean precipitation (*Precip*), deforestation history (*Dist_Time*), distance to smallholder homestead (*Dist_Home*), and distance to roads (*Dist_Roads*) were control variables. The prior for the standard deviation was chosen as t‐distributed, centered on 0, resulting in a slightly regularizing prior while still accounting for extreme parameter values. We sampled 3000 realizations of the posterior distribution using Markov Chain Monte Carlo with two sampling chains running for 2000 iterations and a warm‐up period of 500 iterations. Convergence was verified using the *Rhat* statistic and visual examination of trace plots. We evaluated the model fit based on posterior predictive checks (Figure S1). We performed all modeling through the *brms* package (version 2.17.0, Bürkner 2017) in R (R Core Team 2024) as an interface to the Bayesian inference engine *Stan* (Carpenter et al. 2017).

### 2.5 Analyzing Edge Effects

Using these models, we examined edge effects in the Chaco. First, we assessed the distance at which our response variables reached 90% of their values measured at 2000 m distance, the point beyond which we assumed edge effects were absent (Chaplin‐Kramer et al. 2015), and defined this distance as the penetration depth of the edge effect. Second, we recorded the relative difference of our response variables between the forest edge (i.e., the border to the deforestation plot) and our maximum distance of 2000 m to determine the magnitude of the edge effect. We did this two times, once with and once without considering the age of the deforestation plot (i.e., the year of deforestation). Next, once penetration depth was determined, we mapped it across the remaining forest areas in the Chaco (i.e., buffering all forest areas inward by this distance). We then calculated the percentage of remaining forest areas affected by edge effects, separately for all three structural variables. Lastly, we calculated the estimated aboveground biomass loss through edge effects in the Chaco. To do so, we assumed that the current aboveground biomass in areas not affected by edges represents the potential biomass value of edges, allowing us to derive a relative reduction of aboveground biomass due to the edge effect. We calculated this for all edges across our study region and summarized the total aboveground biomass lost due to edge effects.

## 3 Results

**FIGURE 3:**
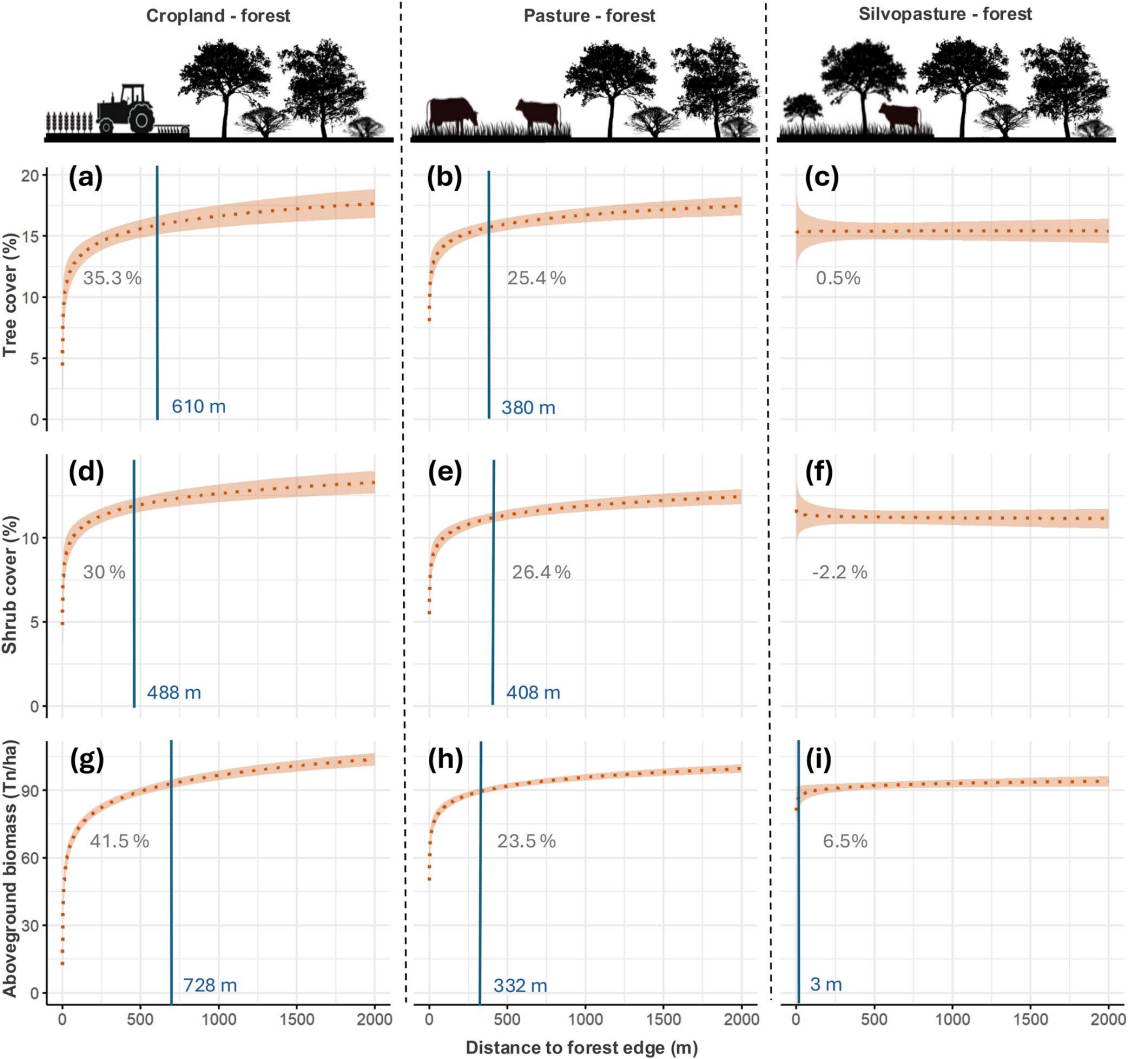
Agriculture‐driven edge effects in the Chaco. Estimated mean conditional effect of the distance to a deforested plot on tree cover (top row), shrub cover (middle), and aboveground biomass (bottom), for three post‐deforestation land uses: Cropland (left column), pasture (centre), and silvopasture (right). In each panel, vertical lines indicate the penetration depth, while numbers below the curves indicate the magnitude of the edge effect.

Our Bayesian models performed well and provided reliable estimates of edge effects and the influence of control variables on forest structure (see Supporting Information for full model outputs and model diagnostics). Our analyses reveal a strong edge effect associated with agricultural expansion on tropical dry forests in the Chaco, consistently across all three forest structural attributes (Figure 3). Generally, these attributes showed logarithmic responses concerning distance from the forest edge, with values being lower near the edge and increasing rapidly at short distances before gradually stabilizing further into the forest. Overall, the shape of the edge effect function was similar across structural parameters, and we observed no clear or consistent differences in effect magnitude or penetration depth (Figure 3).

However, we found marked and consistent differences in edge effects when comparing among different post‐deforestation land uses (Figure 3). Edge effects away from croplands were generally greatest, especially on aboveground biomass with a penetration distance (i.e., distance at which structural attributes reach 90% of the value at 2000 m away from the edge) of 728 m, and a magnitude (i.e., relative difference at the edge and in 2000 m distance) of 41.5% (Figure 3g). For tree and shrub cover, penetration distance was 610 m and 488 m, respectively, and edge effect magnitude was 35% and 30%, respectively (Figure 3a,d). Edge effects away from pastures were much lower, with a penetration distance of 380 m, 408 m, and 332 m for tree cover, shrub cover, and aboveground biomass, respectively, and a magnitude of 25%, 26%, and 23.5%, respectively (Figure 3b,e,h). Silvopasture exhibited negligible edge effects, both in terms of penetration distance and effect magnitude (Figure 3c,f,i).

**FIGURE 4:**
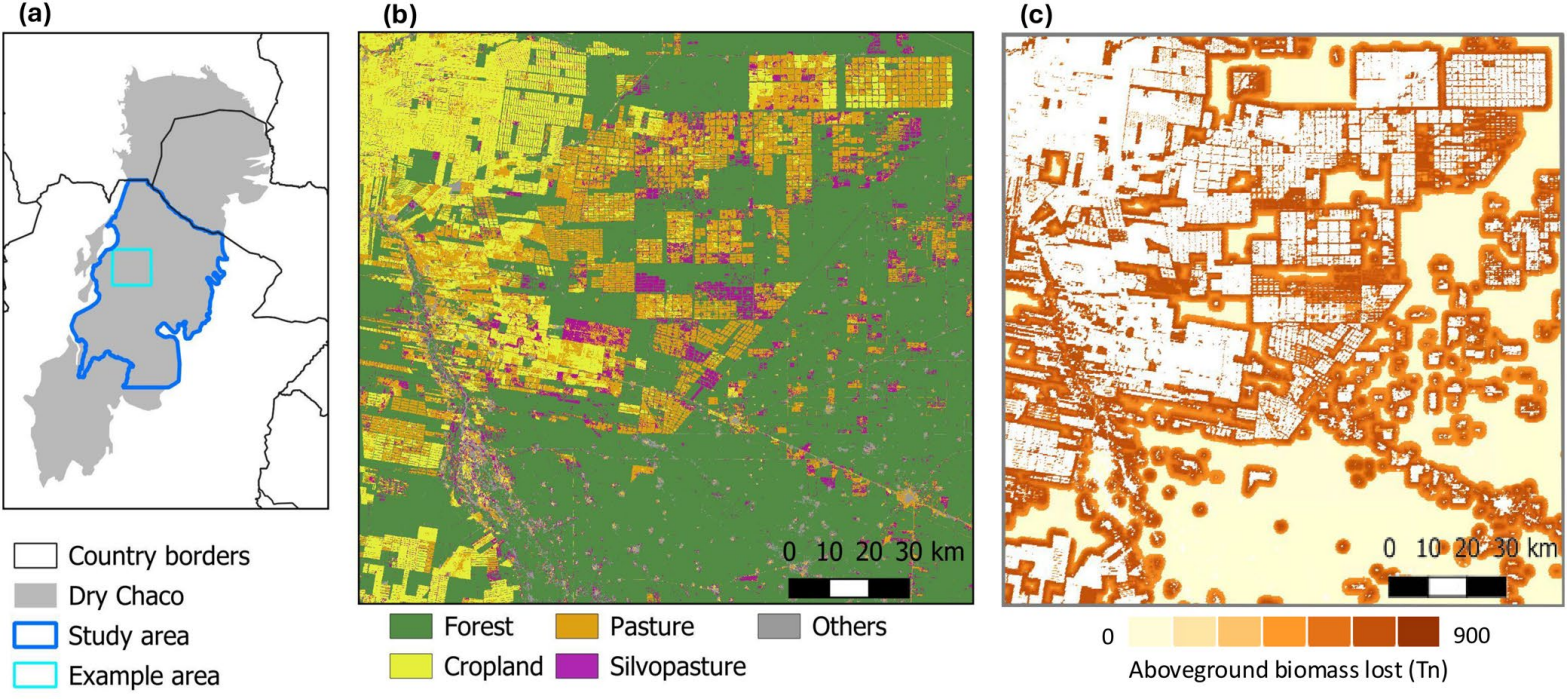
Example of land‐cover patterns (b), and associated edge effects and aboveground biomass lost due to these edge effects (c), in an example area of Dry Chaco (a). Map lines delineate study areas and do not necessarily depict accepted national boundaries.

Combining the estimated penetration distances and magnitudes of these edge effects with our land‐cover maps allowed us to estimate the area impacted by the respective edge effects in our study area (Figure 4). We did this separately for each of our three structural indicators. In terms of aboveground biomass, we estimate that about 3 million ha of forest (18% of the remaining forest in the study area) has a biomass deficit of 10% or more associated with the edge effects of agricultural expansion. Based on this estimation, agriculture edge effects caused the loss of 92.25 million tons of aboveground biomass (Figure 4). This represents roughly 5% of the aboveground biomass of the remaining forests in the study area. For fractional tree and shrub cover, the areas estimated to be affected by edge effects were lower (2.9 Mha and 2.6 Mha, respectively).

**FIGURE 5:**
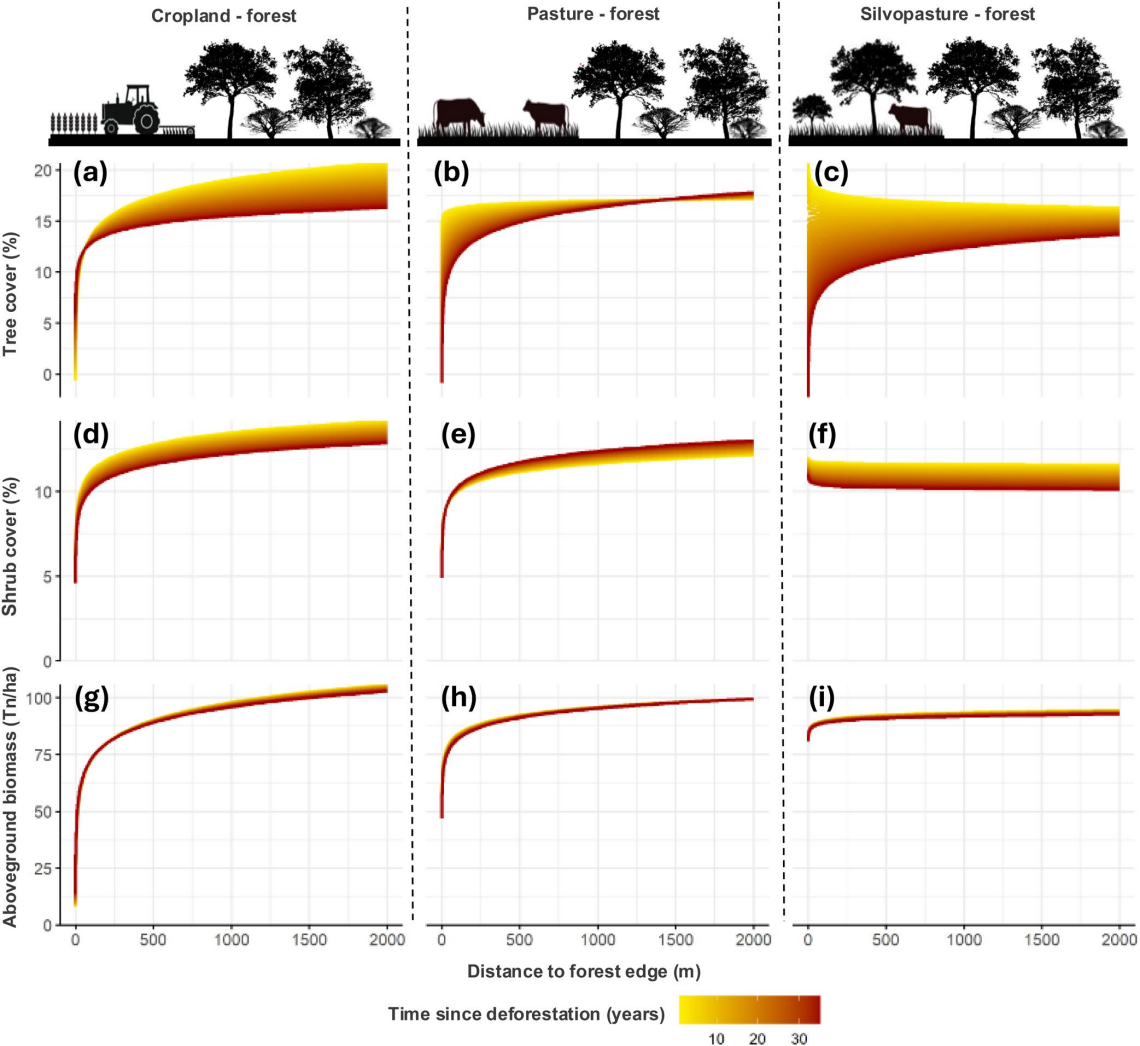
Edge effects over time. Estimated mean conditional effect of the distance to the nearest deforested plot and the time since deforestation on tree cover, shrub cover, and aboveground biomass, considering three different land uses in the plot: Cropland, pasture, and silvopasture.

We also found marked variation in edge effects over time, with generally increasing edge effects as time passed since plots were deforested (Figure 5). For cropland, the overall edge effect magnitude and penetration distance increased over time, especially for tree cover (from 17.5% to 14.8% at a distance of 500 m over 35 years, Figure 5a) and to a lesser extent for shrub cover (from 12.8% to 11.6% at a distance of 500 m over 35 years, Figure 5d). However, we point to the considerable uncertainty connected to the coefficients of the shrub cover models (Table S1). For aboveground biomass, we did not find a considerable temporal effect regarding penetration distance and effect magnitude away from croplands (Figure 5g and Table S1). For pastures, a similar pattern emerged regarding tree cover, with an increasing effect magnitude over time, but penetration distance was shorter and the temporal effect disappeared at about 1–1.5 km (Figure 5b). For shrub cover, the edge effect decreased over time (Figure 5e). Finally, for silvopastures, we found a positive effect (i.e., higher tree cover at closer distances) in early years after deforestation, which however reverted as time progressed (Figure 5c). This is a key additional insight pointing to a potential compensatory effect that could explain why no overall effect was found when time was not considered (Figure 3).

## 4 Discussion

Agricultural expansion into tropical forests is a major sustainability concern, leading to substantial biodiversity losses and carbon emissions. In addition to the direct impact of agriculture expansion and associated forest conversion, forest degradation due to edge effects ensues where agriculture expands but has so far been largely overlooked. This is particularly so for the world's tropical dry forests, which are under‐researched and weakly protected, yet often under higher pressure than rainforests. Here, we focused on the Argentine Dry Chaco, a global deforestation hotspot, to quantify the magnitude and spatial extent of agriculture‐driven edge effects. Using a Bayesian multilevel regression framework, our work provides four major findings. First, we uncover a clear edge effect away from agriculture on forest structure, with penetration distances > 700 m and effect magnitudes > 40%. Second, these edge effects varied strongly depending on the type of agriculture that replaced forests. While cropland and pastures had the strongest edge effects, silvopastures had a much lower and lagged effect. Third, quantifying the spatial footprint of these edge effects, we reveal that 3 Mha of forest (18% of the forest in our study area) suffered from agriculture‐driven edge effects, causing the loss of 92.25 Mt of biomass. Finally, we show how agriculture‐driven edge effects manifest over long periods, with increasing magnitude and penetration distance over several decades, suggesting a degradation debt paid over decades after initial deforestation. Overall, this suggests that agriculture‐driven edge effects are a major, but so far systematically underestimated outcome of agricultural expansion in the tropics.

We found deforestation to cause a strong edge effect, extending more than 700 m into remaining forests. Although there are no other comparable studies from dry forests that we are aware of, our results are in line with studies from rainforests reporting edge effects for structural attributes (e.g., tree cover, tree density, tree mortality). For instance, penetration depth of about 100 m globally (Franklin et al. 2021), up to 200 m in Sumatra (Nguyen et al. 2023), and up to 300 m in the Amazon (Laurance et al. 1998) have been reported. While our penetration depth was longer, a recent pantropical study reported similar edge effects to ours, reaching up to 1500 m penetration depth (Bourgoin et al. 2024). Thus, our results appear plausible and we suggest that shorter penetration depths of earlier work can be explained by less sensitive structural parameters used than the high‐resolution remotely‐sensed indicators employed in our work. Our work differs from prior studies in that we analyzed different strata of the canopy separately. We also found a strong edge effect for shrub cover away from agricultural areas, reaching penetration depths of nearly 500 m. This is an interesting finding because prior work in the Chaco (Lopez de Casenave et al. 1995; Ginzburg 2019) reported an increase in shrub cover at the forest edges. However, these studies only assessed the immediate edge area (e.g., 50 m penetration depth; Ginzburg 2019), highlighting the importance of expanding the spatial scale (up to 2000 m in our case) to reliably detect impact on the shrub strata. Generally, the response of the shrub layer to emerging edges in tropical forests has received little to no attention, with inconsistent results (Franklin et al. 2021). Finally, regarding aboveground biomass, the edge effects we found for the Chaco, with penetration depths > 500 m, are broadly in line but at the upper limit of those reported for rainforests (Chaplin‐Kramer et al. 2015; Ordway and Asner 2020; Silva Junior et al. 2020), suggesting a potentially larger effect in dry forests.

Most importantly, our results reveal major differences in edge effects relative to the post‐deforestation land use, in terms of penetration distance, magnitude, and time period over which edge effects manifest (Figures 3 and 5). To our knowledge, this is the first comparative, regional‐scale assessment of post‐deforestation land use on edges, highlighting that a key determinant of edge effects has so far been largely overlooked. We found particularly strong edge effects for industrialized agriculture, which in the Chaco leads to a full conversion of forest. Edge effects were markedly larger for cropland compared to pastures, particularly for aboveground biomass and tree cover, which in the case of the Chaco can be explained by substantial pesticide drift, given that agribusiness cropping is largely based on no‐till, pesticide‐resistant soy/corn production, where intensive and often untargeted (e.g., by airplanes) pesticide applications occur. Interestingly, for all structural metrics, silvopastures only had a weak, in many cases hardly noticeable, edge effect (Figure 3). This is likely explained by the relatively small physiognomic contrast between forest and silvopastures, with the latter containing both large trees as well as often also small trees and shrubs (Fernández et al. 2024). Overall, this aligns with other work suggesting that silvopastoral systems could lessen the trade‐off between livestock production and biodiversity and carbon stocks considerably (Mastrangelo and Gavin 2014; Macchi et al. 2019; Law et al. 2021). We note, however, that this pattern changes when temporal dynamics are considered, as discussed below. A key finding of our work is that a large area (up to 3 Mha or 18% of remaining forests) is likely affected by the agriculture‐driven edge effects we quantify. This translates into carbon emissions of about 92 Mt from reduced aboveground biomass (4.95% of the remaining AGB), which is equivalent to approximately 15% of the losses caused by deforestation between 1985 and 2013 in the entire Dry Chaco (Baumann et al. 2017). This represents a considerable, and so far unaccounted for, source of carbon emissions linked to agriculture‐driven deforestation. Similar dynamics are likely for other tropical deforestation frontiers (Pendrill et al. 2019; Buchadas et al. 2022), suggesting that edge effects and the ‘bleeding’ of carbon losses into forests adjacent to deforested areas should be incorporated into global, regional, and national emission assessments (Chaplin‐Kramer et al. 2015).

Our final main finding highlighted that deforestation creates long‐lasting legacies in terms of edge effects, given that both the magnitude and penetration depth increased considerably over long periods following initial deforestation (Figure 5). Worryingly, we found no evidence of stabilization even after 35 years of edge creation. This means that the current forest structure at the forest/agriculture interface cannot be seen to represent a stationary state, suggesting an unpaid ‘degradation debt’. We note that this effect was mainly observable for tree cover, but not or less so for shrub cover and aboveground biomass, possibly because tree‐cover models had a higher accuracy than the shrub‐cover and aboveground‐biomass models (Baumann et al. 2018; Pötzschner et al. 2022). Our results are well in line with other time‐lagged responses (Lira et al. 2019; Chen et al. 2023). For instance, extinction debt has been shown for birds and mammals (Semper‐Pascual et al. 2021) and plants (Herrero‐Jáuregui et al. 2022) for the Dry Chaco. In work on tropical forest degradation, however, such time lags have so far received limited attention. Furthermore, this analysis also uncovered that edge effects for silvopastures do develop over time, a pattern that would remain masked for snapshot analysis (Figure 3). The lagged emergence of edge effects (after about 20 years) is well in line with reports on tree‐cover loss from silvopastures due to inappropriate shrub control measures that harm trees (Fernández et al. 2020), suggesting that even for silvopastures a degradation debt may exist. We used high‐resolution indicators for deforestation, land use, and forest structure, and our Bayesian multi‐level regression framework performed well. Nevertheless, a few limitations need to be discussed. First, we considered only the distance to the nearest edge per sampling point, which means we did not account for the possible compound effect of multiple edges affecting a plot (Harper et al. 2007; Ries et al. 2017). This is likely most relevant for small patches, which are abundant in some parts of the region (Camba Sans et al. 2021; Kliger and Ginzburg 2022). Second, we considered only the current post‐deforestation land use, although initial pastures can sometimes transition to cropland after deforestation (Baumann et al. 2022). We note, however, that the edge effects were fairly similar for pastures and cropland. Third, the silvopasture category might include some areas where deforestation is ongoing or where land has been partially cleared for resale, but land use has not ensued. This would introduce noise in our results and cause an underestimation of the edge effect associated with silvopastures.

Our work has major implications for conservation and land‐use planning in tropical dry forests and active deforestation frontiers. First, to maintain long‐term forest integrity, it is important to minimize forest/agriculture edges and to ensure the adequate protection of large, contiguous patches of forest. Second, our work emphasizes the importance of a biodiversity‐friendly agricultural matrix (Fletcher Jr et al. 2024), with silvopastures as a possibly much less detrimental system compared to other land uses (Law et al. 2021; Fernández et al. 2024). Importantly, connectivity among large forest patches should be maintained (Kliger and Ginzburg 2022; Torrella et al. 2018). However, we highlight that even degraded forest strips and small forest patches contribute to connectivity (Camba Sans et al. 2021; Kliger and Ginzburg 2022) and are important for biodiversity (Torrella et al. 2011; Semper‐Pascual et al. 2021; Herrero‐Jauregui et al. 2022). Third, agricultural management should be adjusted to minimize pesticide drift into adjacent forests (Ferreira et al. 2023), for instance, through buffer zones, more targeted application, or application only when there is no wind (Cederlund 2017). Agriculture expansion is a major threat to tropical dry forests globally. Here, we show that in addition to forest conversion, agricultural expansion results in large areas of forest degradation away from the forest/agriculture edge. Our findings indicate that these effects are substantial in both magnitude and extent, and that they unfold over decades, representing an overlooked but important mechanism of forest degradation. Our study focuses on the Argentine Dry Chaco, but similar processes are likely occurring across agriculture frontiers in tropical dry forests and other forests worldwide. Recognizing and addressing agriculture‐driven edge effects is therefore critical to better account for the full environmental costs of agricultural expansion.

## Author Contributions

**Sebastián Torrella:** conceptualization, investigation, writing – original draft, methodology, writing – review and editing, data curation. **Matthias Baumann:** conceptualization, writing – review and editing, software, formal analysis, data curation, methodology, investigation. **Marie Pratzer:** software, formal analysis, data curation. **Sebastián Aguiar:** writing – review and editing. **María Piquer‐Rodríguez:** conceptualization, writing – review and editing. **Rubén Ginzburg:** conceptualization, writing – review and editing. **Gregorio Gavier Pizarro:** conceptualization, writing – review and editing. **Tobias Kuemmerle:** conceptualization, funding acquisition, writing – review and editing, methodology, project administration, supervision.

## Funding

This work was supported by H2020 European Research Council, 101001239. Universidad de Buenos Aires, 0020220400286BA.

## Conflicts of Interest

The authors declare no conflicts of interest.

## Supporting information


**Table S1:** Estimations for the effects of model coefficients. Effects of distance to the nearest plot (Distance) and age of the nearest plot (Age) are presented by land use (group) level, while effects of covariates are for the global models. Covariates are annual mean temperature (Temp), annual mean precipitation (Precip), an indicator of the distance to the nearest plot over time (Dist_Time), distance to the nearest smallholder homestead (Dist_Home) and distance to the nearest road (Dist_Roads). The table shows the posterior mean as a measure of central tendency and the standard error as a measure of uncertainty. Please note that the response variable has been log‐transformed and all variables have been standardized in the model. White cells represent models that do not account for edge age, whereas gray cells represent models that include the interaction between distance and edge age.
**Figure S1:** Posterior predictive distributions and observed data distribution for the tree cover, shrub cover and aboveground biomass models (For the models that doesn't include edge age as factor).
**Figure S2:** Posterior predictive distributions and observed data distribution for the tree cover, shrub cover and aboveground biomass models including edge age as factor.

## Data Availability

The data that support the findings of this study are openly available in Zenodo at https://doi.org/10.5281/zenodo.16647865, reference number 16647865.
