# Supplementary material for: Quantifying the Spatial Footprint of Agriculture‐Driven Edge Effects in a Global Deforestation Hotspot
Source: Glob Chang Biol. 2026 Feb 10;32(2):e70737. doi: 10.1111/gcb.70737 (PMC12891297; doi:10.1111/gcb.70737)
Supplement: Supplementary file 1 — Table S1: Estimations for the effects of model coefficients. Effects of distance to the nearest plot (Distance) and age of the nearest plot (Age) are presented by land use (group) level, while effects of covariates are for the global models. Covariates are annual mean temperature (Temp), annual mean precipitation (Precip), an indicator of the distance to the nearest plot over time (Dist_Time), distance to the nearest smallholder homestead (Dist_Home) and distance to the nearest road (Dist_Roads). The table shows the posterior mean as a measure of central tendency and the standard error as a measure of uncertainty. Please note that the response variable has been log‐transformed and all variables have been standardized in the model. White cells represent models that do not account for edge age, whereas gray cells represent models that include the interaction between distance and edge age. Figure S1: Posterior predictive distributions and observed data distribution for the tree cover, shrub cover and aboveground biomass models (For the models that doesn't include edge age as factor). Figure S2: Posterior predictive distributions and observed data distribution for the tree cover, shrub cover and aboveground biomass models including edge age as factor. [file GCB-32-e70737-s001.docx]

Supplementary Information

**Estimated model coefficients**

The control variables had varying effects on the response variable (Supplementary Table 1). While climatic variables (temperature and precipitation) meaningfully contributed to predicting shrub cover and aboveground biomass, distance to the nearest smallholder homestead had a strong effect on tree cover, but not on the other response variables. We found that [Dist_time] and distance to the nearest road did not meaningfully contribute to predicting forest structure in our models.

***Supplementary Table 1.*** *Estimations for the effects of model coefficients. Effects of distance to the nearest plot (Distance) and age of the nearest plot (Age) are presented by land use (group) level, while effects of covariates are for the global models. Covariates are annual mean temperature (Temp), annual mean precipitation (Precip), an indicator of the distance to the nearest plot over time (Dist_Time), distance to the nearest smallholder homestead (Dist_Home) and distance to the nearest road (Dist_Roads). The table shows the posterior mean as a measure of central tendency and the standard error as a measure of uncertainty. Please note that the response variable has been log-transformed and all variables have been standardized in the model. White cells represent models that do not account for edge age, whereas gray cells represent models that include the interaction between distance and edge age.*


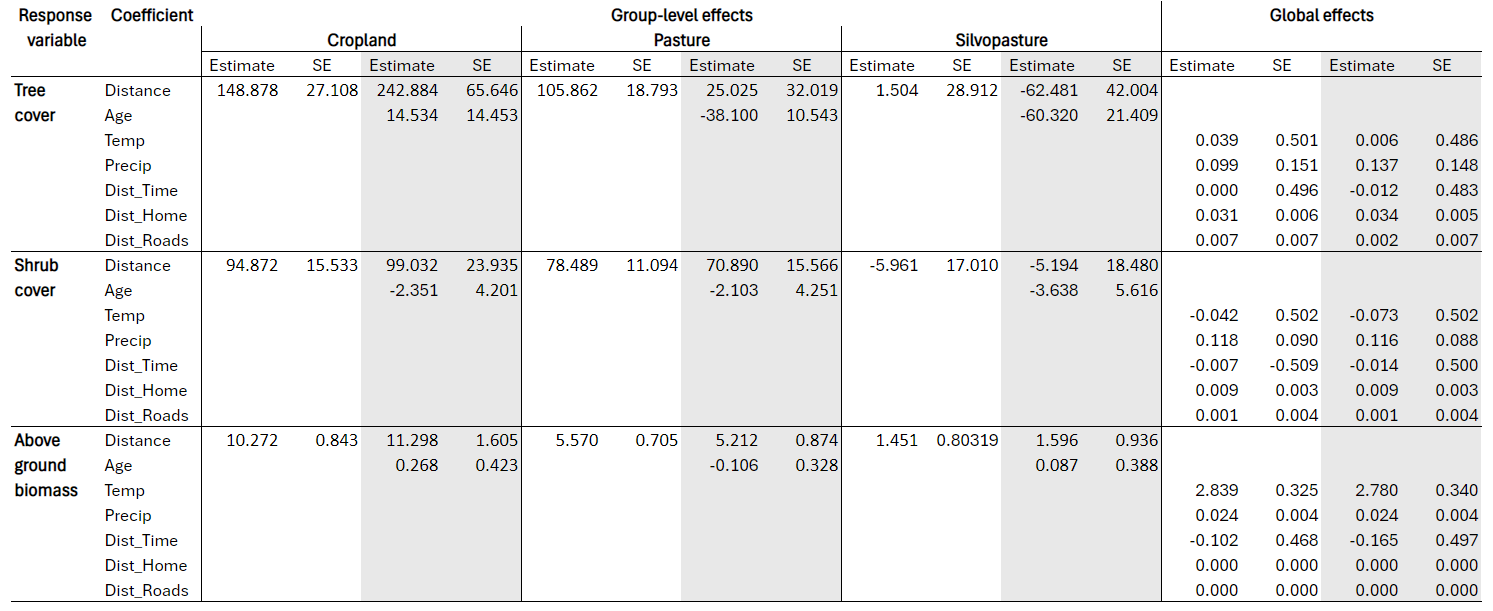


**Model diagnostics and predictive performance**

Applied models configuration effectively performed in predicting the response variables (tree cover, shrub cover and aboveground biomass). The sampling process demonstrated its efficacy, as evidenced by the values of R̂ closely approximating one for all parameters. Quantities of interest suggest that the chain successfully converged to the stationary distribution, and the trace plots reveal a good level of mixing (van de Schoot et al. 2014). To gauge the predictive power of the models concerning the response variables, we conducted a thorough comparison between the posterior predictive distribution and the distribution of the observed data. These two distributions exhibited a high degree of alignment (Supplementary Figure 1 and 2).


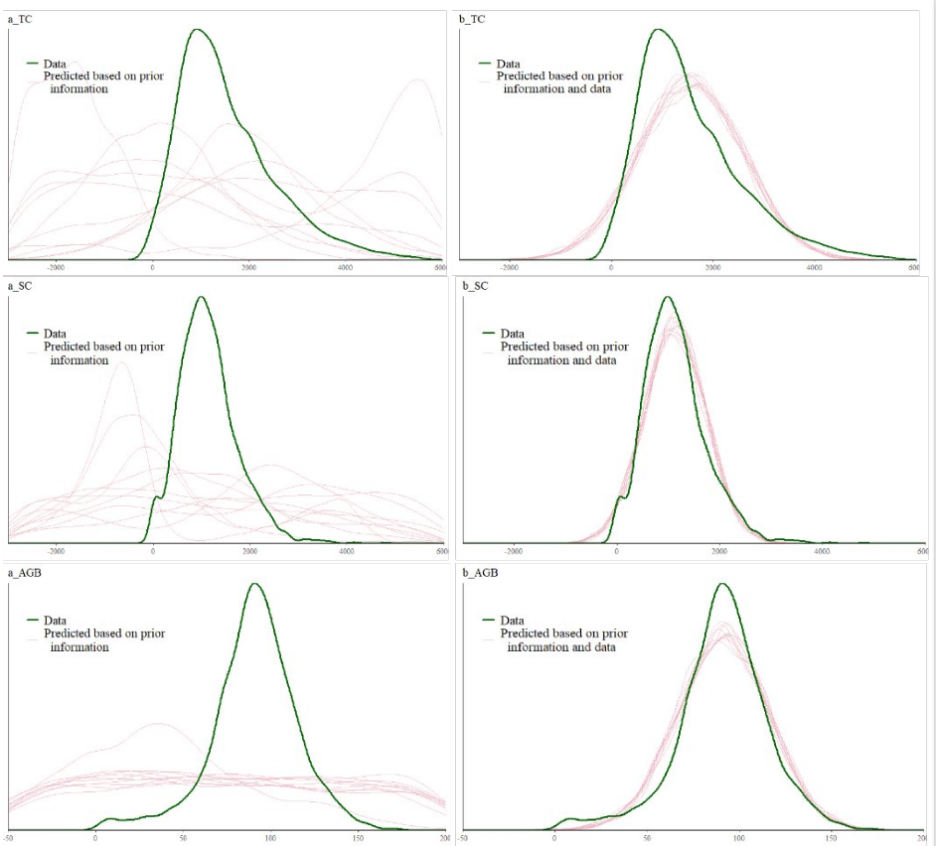
**Supplementary figure 1.** Posterior predictive distributions and observed data distribution for the tree cover, shrub cover and aboveground biomass models (For the models that doesn't include edge age as factor).


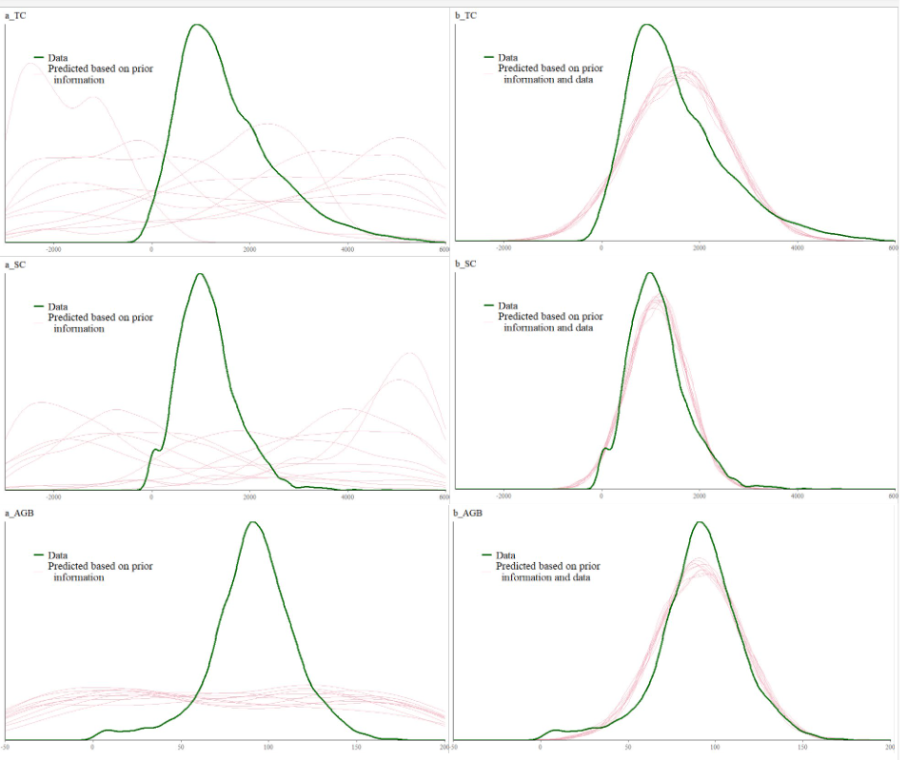
**Supplementary figure 2.** Posterior predictive distributions and observed data distribution for the tree cover, shrub cover and aboveground biomass models including edge age as factor.

**References**

van de Schoot, R., Depaoli, S., King, R., Kramer, B., Martens, ¨ K., Tadesse, M.G., Vannucci, M., Gelman, A., Veen, D., Willemsen, J., Yau, C., 2021. Bayesian statistics and modelling. Nat. Rev. Methods Prim. 1
